# Successful treatment of rectovaginal fistula and rectal stenosis due to perianal Crohn’s disease by dual-port laparoscopic abdominoperineal resection: a report of two cases

**DOI:** 10.1186/s40792-016-0211-0

**Published:** 2016-08-27

**Authors:** Fumihiko Matsuzawa, Shigenori Homma, Tadashi Yoshida, Susumu Shibasaki, Nozomi Minagawa, Tatsushi Shimokuni, Hideyasu Sakihama, Hideki Kawamura, Norihiko Takahashi, Akinobu Taketomi

**Affiliations:** Department of Gastroenterological Surgery I, Hokkaido University Graduate School of Medicine, North 15, West 7, Kita-Ku, Sapporo, Hokkaido 060-8638 Japan

**Keywords:** Rectovaginal fistula, Rectal stenosis, Perianal Crohn’s disease, Reduced port surgery, Dual-port laparoscopic abdominoperineal resection

## Abstract

**Background:**

The incidence of rectovaginal fistula in women with Crohn’s disease has been reported to be 3–10 %. Although rectovaginal fistulas can be managed medically and surgically, they have high rates of recurrence and complications. Rectal stenosis is another condition that occurs due to perianal Crohn’s disease. A novel, minimally invasive procedure, dual-port laparoscopic abdominoperineal resection using a multichannel port, has been shown effective in patients with lower rectal cancer and patients with medically uncontrolled ulcerative colitis. This report describes the use of the same method for two patients with Crohn’s disease-related rectovaginal fistula and rectal stenosis.

**Case presentation:**

The first patient, a 22-year-old woman, was diagnosed with rectovaginal fistula and rectal stenosis due to perianal Crohn’s disease 2 years earlier. Induction therapy with infliximab and endoscopic balloon dilatation did not improve her symptoms. The second patient, a 33-year-old woman, was also diagnosed with rectovaginal fistula and rectal stenosis due to perianal Crohn’s disease, and medical treatment was also unsuccessful. Both patients underwent dual-port laparoscopic abdominoperineal resection using a multichannel port, with no perioperative and postoperative complications.

**Conclusion:**

These findings show that this reduced port method can be used to successfully treat patients with Crohn’s disease-associated rectovaginal fistula and rectal stenosis.

## Background

Fistulas are abnormal tracts arising from mucosal ulcerations in the gastrointestinal tract that have progressed to penetrate other structures [1]. The cumulative incidence of fistulas in patients with Crohn’s disease (CD) has been reported to be 33 % after 10 years and 50 % after 20 years, with 83 % of all fistula episodes requiring surgery [2]. The incidence of rectovaginal fistulas (RVF) in women with CD has been reported to be approximately 3–10 %. Although medical and surgical management techniques have been described, these fistulas are difficult to manage because of their high rates of recurrence and complications. In addition to RVF, women with CD can also experience rectal stenosis, a major complication that is also difficult to manage [3].

We have described the successful use of laparoscopically assisted anterior resection, using a single incision and one additional port, for patients with rectal cancer [4]. Based on this technique, we have developed a novel, minimally invasive procedure, dual-port laparoscopic abdominoperineal resection using a multichannel port, and shown its effectiveness in patients with lower rectal cancer and patients with medically uncontrolled ulcerative colitis [5, 6]. This report documents the use of the same method in two patients with CD-related RVF and rectal stenosis.

## Case presentation

### Patients

**Fig. 1:**
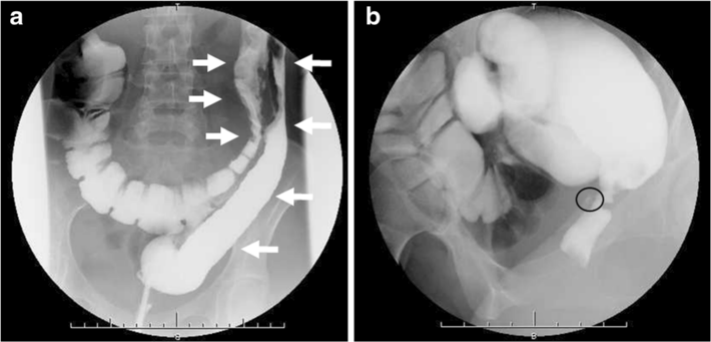
Preoperative lower gastrointestinal series of patient 1, showing stenosis and the disappearance of haustra in the transverse and descending colon (*white arrow* in **a**) and the rectovaginal fistula (*black circle* in **b**)

**Fig. 2:**
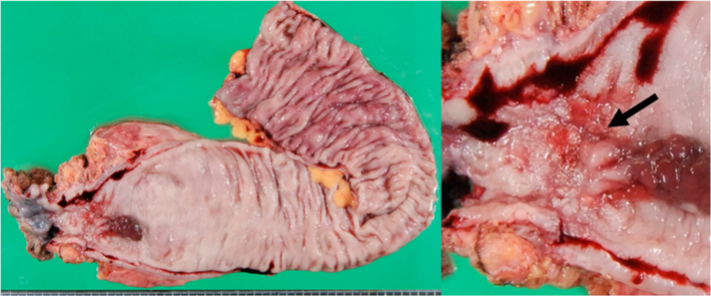
View of the resected specimen from patient 2, showing a fistula from the rectum to the vagina (*black arrow*)

A 22-year old woman was diagnosed with colonic CD 6 years earlier. For 4 years, she experienced fluctuating disease, with periods of remission and recurrence. She was initially treated with maximal doses of 5-aminosalicylate (5-ASA), and corticosteroids, but later switched doses to azathioprine. Two years earlier, she started to pass gas from her vagina, and she was diagnosed with RVF due to CD. She was started on induction therapy using infliximab, but her symptoms did not improve. She also had a 2-year history of intestinal obstruction due to CD-associated rectal stenosis. She underwent endoscopic balloon dilatation to treat the stenosis, but experienced no improvement. Because of her inability to take nutrition through her intestinal tract, she was started on total parenteral nutrition. She consulted our department for the treatment of RVF and repeat intestinal obstruction. A lower gastrointestinal series showed the disappearance of haustra, along with stenosis in the transverse and descending colon (Fig. 1a), rectal stenosis and RVF (Fig. 1b). Dual-port laparoscopic abdominoperineal resection, from the transverse colon near the splenic flexure to the rectum, was performed. The resected specimen showed both the RVF and rectal stenosis (Fig. 2). The patient experienced no perioperative or postoperative complications. She was discharged 14 days after surgery and has not experienced any recurrence of CD for 3 years.

A 33-year old woman was diagnosed with small intestinal and colonic CD 14 years earlier. Six years ago, she underwent ileocecal resection for stenosis of the ileum. After surgery, she was started on infliximab but had to discontinue treatment because of the side effects of that drug. Three years ago, she started to pass gas from her vagina, and she was diagnosed with RVF due to CD. Medical therapy was unsuccessful, and she consulted our department. A preoperative lower gastrointestinal series showed the RVF, along with stenosis and the disappearance of haustra in the transverse and descending colon. She underwent the same operation as the first patient, with the same extent of resection. Pathological examination showed a fistula from the rectum to the vagina and stenosis from the transverse colon to the rectum. She did not experience any perioperative or postoperative complications. She was discharged 15 days after surgery and has not experienced recurrence of CD for 4 years.

### Operative procedures

**Fig. 3:**
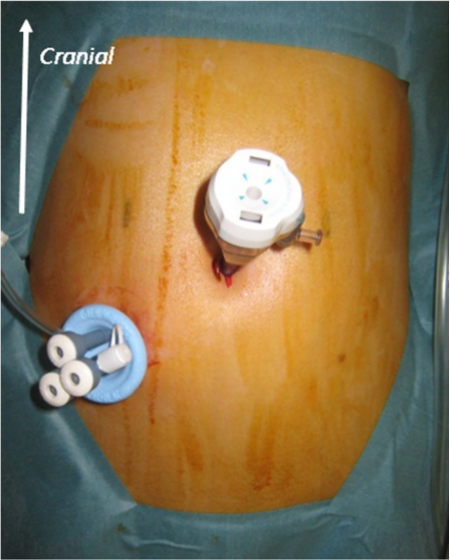
Insertion of a multichannel port at the colostomy site through a 25-mm skin incision in the right lower quadrant. A 5-mm trocar was inserted via the umbilicus

**Fig. 4:**
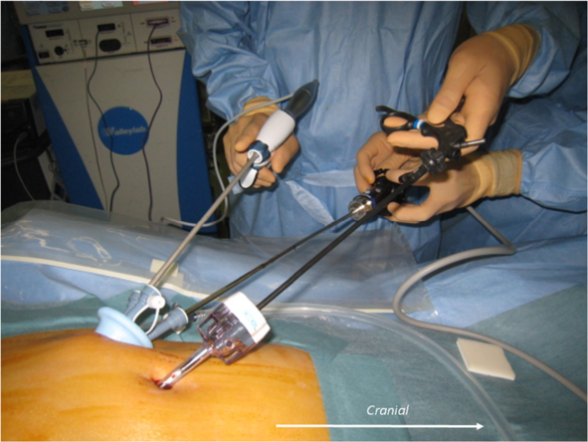
Positioning of the surgeon and assistant on the patient’s right side

**Fig. 5:**
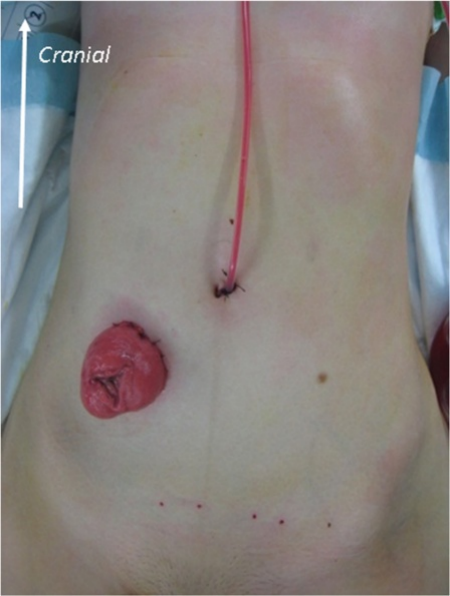
Creation of a colostomy at the site of the multichannel port and placement of the drain through the umbilicus

A multichannel port was inserted through a 25-mm skin incision in the right or left lower quadrant at the colostomy site. The colostomy site was determined by the preoperative marking, regardless of the surgical procedure. The choice of multichannel port site was based on the length of colon to be resected. An additional 5-mm trocar for the left hand was inserted at the umbilicus (Fig. 3). The surgeon and the assistant were positioned on the patient’s right side (Fig. 4). For anterior dissection, the uterus was suspended by two stitches of 3–0 monofilament thread penetrating the left and right lower abdomen near the pubic bone. The assistant retracted the rectosigmoid ventrally using a curved grasper, and the peritoneum was incised using an ultrasonic coagulation incision device from the level of the sacral promontory to the base of the inferior mesenteric artery. After identification of the left ureter, the vascular pedicle was lifted vertically by the assistant. The inferior mesenteric artery was isolated and ligated 1 cm distal to the aorta. The inferior mesenteric vein was also ligated. Pelvic dissection was performed along the presacral avascular plane down to the pelvic floor and the top of the anal canal. The rectum was completely mobilized to the level of the levator ani muscle. The oral side of the intestine, which was free of the mucosal disorder due to CD, was cut off. Perineal dissection was performed circumferentially to the pelvic cavity. The specimen was retrieved through the perineal wound. A drain was placed from the umbilicus into the pelvic bottom, and a colostomy was fashioned at the site of the multichannel port (Fig. 5).

## Discussion

This study reports the performance of dual-port laparoscopic abdominoperineal resection using a multiple port method. Neither patient experienced any perioperative or postoperative complications. In contrast, the rates of complications were reported to be much higher in patients with CD-related RVF who underwent conventional proctectomy, with 35 % experiencing delayed perineal wound healing, 17 % having intra-abdominal sepsis, and 15 % experiencing stomal complications [7]. Another series reported delayed or failed perineal wound healing in almost 50 % of patients [8]. The novel, minimally invasive procedure described here, dual-port laparoscopic abdominoperineal resection using a multiple port, has been found effective in patients with lower rectal cancer [5] and in patients with medically uncontrolled ulcerative colitis [6]. This procedure was completed successfully in all patients, without any intraoperative complications, and all postoperative outcomes were satisfactory.

This report describes our use of this surgical method in two patients with CD-related RVF and rectal stenosis. The abdominal cavity was approached using two small incisions, with a multichannel port placed at the colostomy site through a 25-mm skin incision in the lower quadrant and a 5-mm trocar inserted via the umbilicus for postoperative placement of a drainage tube. Neither patient experienced any intraoperative or postoperative complications. We recently reported that the postoperative neutrophil count was lower after SLIS +1 port laparoscopy-assisted than after conventional laparoscopy-assisted anterior resection for rectal cancer [4]. Furthermore, the former group experienced a significant difference in body temperature on postoperative day 1, indicating a lower degree of inflammation. Our findings suggest that this procedure, involving small incisions and minimal invasiveness, may reduce the risk of complications and benefit not only patients with rectal cancer and ulcerative colitis but also patients with CD-related RVF and rectal stenosis.

CD is the second most common cause of RVF after obstetrical trauma. The incidence of RVF in women with CD is approximately 3–10 %. RVF may cause significant clinical distress and social embarrassment. RVFs are extremely difficult to close medically [9], often leaving surgery as the only option [10]. Medical treatments have included antibiotics, corticosteroids, and immunosuppressants, but these agents are associated with low rates of long-term symptom control and unacceptably high rates of recurrence [11]. Infliximab, a monoclonal antibody to tumor necrosis-α (TNF-α), is a major advance in the treatment of fistulizing CD disease and has completely altered treatment strategies for perianal disease [12]. However, analysis of the results of the ACCENT II (A CD Clinical trial Evaluating infliximab in a New long-term Treatment regimen in patients with fistulizing CD) trial found that 56 % of patients on maintenance therapy with infliximab experienced RVF recurrence [10]. Another study showed that response to infliximab differed among patients with different types of CD fistula [13]. The closure rate after 4 to 6 weeks of treatment was 76 % for all external CD fistulas, but only 14 % for CD-associated RVF [13].

Patients who cannot be managed medically or are resistant or intolerant to infliximab can be managed surgically. Proctectomy was performed initially because of the high recurrence rate of CD-related RVF and difficulties treating rectal stenosis. To date, there have been no prospective, randomized, controlled trials assessing methods for the surgical correction of CD-related RVF [14]. Transvaginal, perineal, and transanal approaches, with or without transabdominal mobilization, can be used for local repair. Fecal diversion remains a problem, but protecting the fistula repair with a diverting stoma was reported to improve healing and reduce recurrence [15]. However, even with a diverting stoma, the cure rate remains less than satisfactory. The rates of recurrence of CD-related RVF have been reported to range from 25 to 50 %, higher than the recurrence rates of other CD fistulas [16–20]. Furthermore, most studies have considered only short-term outcomes. For example, of 12 patients with CD-related RVF who underwent local repairs, 7 (58 %) showed recurrence [16]. Proctectomy is still considered the only radical cure for Crohn’s-related RVF.

Both patients in this report presented with RVF and rectal stenosis, with the latter causing repeat intestinal obstruction. Rectal strictures due to CD are as difficult to treat as RVF. For example, 66 % of patients with perianal CD and rectal strictures required a permanent stoma, with multivariate analysis showing that rectal stricture was independently predictive of the need for a permanent stoma [3].

This report has certain limitations. First, both patients had low degrees of adhesion, allowing conventional laparoscopic surgery. Second, this operation required high operative skill of the entire surgical team. The surgeon and team in this study had experience with over 500 laparoscopic colorectal resection procedures, including reduced port surgery for colorectal diseases including colorectal cancer.

## Conclusions

CD-related RVF and rectal stenosis are difficult to manage medically and surgically. Dual-port laparoscopic abdominoperineal resection using a multichannel port, when performed by experienced surgeons, may be useful for selected patients with these conditions.

## Consent

Written informed consent was obtained from both patients for publication of this case report and any accompanying images. A copy of the written consent is available for review by the Editor-in-Chief of this journal.

## Abbreviations

CD: Crohn’s disease
RVF: rectovaginal fistulas.
